# Prediction of diabetes mellitus induced by steroid overtreatment in adrenal insufficiency

**DOI:** 10.1038/s41598-022-04904-w

**Published:** 2022-01-18

**Authors:** Valentina Guarnotta, Laura Tomasello, Carla Giordano

**Affiliations:** Dipartimento di Promozione della Salute, Materno-Infantile, Medicina Interna e Specialistica di Eccellenza “G. D’Alessandro” (PROMISE), Sezione di Malattie Endocrine, del Ricambio e della Nutrizione, Università di Palermo, Piazza delle Cliniche 2, 90127 Palermo, Italy

**Keywords:** Diseases, Endocrinology

## Abstract

To assess the differences between patients with normal glucose tolerance (NGT) and prediabetes/diabetes mellitus (DM) in secondary adrenal insufficiency (SAI). We cross-sectionally evaluated 102, out of a total of 140, patients with SAI, who were on hydrocortisone (HC) (n = 50) and cortisone acetate (n = 52) replacement therapy. Clinical, anthropometric, and metabolic parameters were compared in patients with NGT (n = 60) and DM (n = 42). Patients with prediabetes/DM have a more marked family history of DM (*p* = 0.002), BMI (*p* < 0.001), higher waist circumference (*p* < 0.001), total cholesterol (*p* = 0.012), LDL-cholesterol (*p* = 0.004), triglycerides (*p* = 0.031), fasting glucose (*p* = 0.002), fasting insulin (*p* = 0.035), glutamate pyruvate transaminase (*p* = 0.018), HOMA-IR (*p* = 0.039), area under curves of glucose (*p* = 0.001) and insulin (*p* = 0.002), HbA1c (*p* < 0.001), Visceral adiposity index (VAI) (*p* = 0.038) and lower ISI-Matsuda (*p* = 0.008) and oral disposition index (*p* < 0.001) than patients with NGT. Multivariate analysis showed that family history of DM and VAI are independent predictive factors for DM in patients with SAI. Family history of DM and VAI can be predictors of the development of DM in patients with SAI and need to be investigated during steroid replacement therapy. Interestingly, the type and the dose of replacement steroid do not impact on diabetes mellitus.

## Introduction

Secondary adrenal insufficiency (SAI) is characterized by the failure of pituitary disease to produce ACTH. Rarely, it is isolated, while more frequently it is associated with other pituitary deficiencies such as hypothyroidism, hypogonadism and growth hormone deficiency (GHD). Subjects with SAI have higher mortality than the general population without SAI. The main causes of mortality are represented by cardiovascular disease, tumours and infections^1^.

Cardiovascular mortality is due to cardiovascular risk factors such as diabetes mellitus, dyslipidaemia and arterial hypertension^2^. Diabetes mellitus is quite frequent in patients with SAI. Excluding autoimmune diabetes mellitus, diabetes mellitus in SAI has been recognized to be due to inappropriate glucocorticoid (GC) replacement therapy^3^. Therapeutic steroid replacement management of SAI consists in cortisone acetate at the daily dose of 25–30 mg and hydrocortisone (HC) at the daily dose of 15–25 mg administered in two or three doses or, as an alternative, when cortisone acetate and/or HC are not available, prednisolone once or twice daily at the dose of 3–5 mg/day^4^. As reported in many studies, patients overtreated for a long period with conventional steroids develop frequently diabetes mellitus and dyslipidaemia, while novel formulations have no impact on metabolism^5–9^. Discordant reports are available between the association of high doses of cortisone replacement therapy and cardiovascular mortality, higher risk of diabetes mellitus and other comorbidities^10–12^. Currently, the factors involved in the development of diabetes mellitus in patients with SAI treated with conventional steroids have not been fully investigated.

The primary aim of the current study was to assess the prevalence of glucose tolerance defects in a population of patients with SAI and to evaluate the differences between patients with normal glucose tolerance (NGT) and patients with prediabetes and diabetes mellitus. The secondary aim was to identify predictive factors for the development of diabetes mellitus in order better to personalize steroid replacement treatment.

## Materials and methods

### Study participants

We evaluated data from 102 consecutive patients with SAI due to hypopituitarism, out of a total of 140 patients who were on conventional GC treatment. The patients were consecutively referred to the Division of Endocrinology of Palermo University from January 2010 to December 2020. All patients had a disease duration of at least 5 years. Inclusion criteria were the following: age 18–75 years, diagnosis of SAI, ongoing daily conventional GC treatment for at least 5 years, stable replacement dose with levo-thyroxine, testosterone, estrogen and GH for at least 3 months before inclusion and during follow-up, stable dose of cortisone acetate and HC for at least 6 months or more, before inclusion in the study. Exclusion criteria were pregnancy, lactation, primary adrenal insufficiency and treatment with dual-release hydrocortisone.

Fifty patients were on HC replacement treatment, while 52 were on cortisone acetate therapy.

SAI was diagnosed as recommended by international guidelines^4^. The metabolic syndrome was diagnosed according to the NCEP ATP III criteria, whereas diabetes mellitus and prediabetes were diagnosed according to the ADA criteria^13,14^. We defined visceral obesity as the presence of waist circumference over 102 cm in males or 88 cm in females.

Overall, 60 patients had normal glucose tolerance, while 42 had prediabetes/diabetes mellitus. Patients with diabetes mellitus were all on metformin treatment with a ranging dose from 1000 to 2550 mg/day. Among patients with arterial hypertension, 6 were treated with calcium channel blockers, 2 with furosemide and 5 were treated with angiotensin Receptor Blockers (ARBs).

Patients with hypothyroidism were treated with levo-thyroxine at the average dose of 1 mcg/kg. Patients with GHD were treated with somatotropin at the average dose of 0.4 mg/day. Males with hypogonadism were treated with an average injected monthly dose of testosterone enanthate 250 mg. Premenopausal females were treated with a low dose of estrogen and progesterone therapy. No history of chronic GC use before substitutive treatment was known for patients with SAI.

We defined patients on high doses of steroid replacement treatment ≥ 37.5 mg/day of cortisone acetate and 30 mg/day of HC.

This study was carried out in accordance with the recommendations of the Paolo Giaccone Policlinico ethics committee with written informed consent from all subjects, in accordance with the Declaration of Helsinki. The protocol was approved by the Paolo Giaccone Policlinico ethics committee.

### Study design

In this cross-sectional, real-life study we investigated the effects of cortisone replacement therapy in patients with SAI. Patients were subdivided into two groups: NGT (normal glucose tolerance) and prediabetes/diabetes mellitus. Body mass index (BMI), waist circumference (WC) measured at the midpoint between the lower rib and the iliac crest, and waist/hip ratio (WHR) were evaluated. Lipid profile [total, high-density lipoprotein (HDL) and low-density lipoprotein cholesterol (LDL), triglycerides], haemoglobin A1c (HbA1c), glycaemia and serum insulin were extracted after overnight fasting. Metformin was suspended 48 h before the biochemical assays.

Oral glucose tolerance test was carried out by measuring plasma blood glucose and insulin levels every 30 min for 2 h after a 75-g of oral glucose load.

Insulin sensitivity was estimated indirectly using basal insulin and glucose values to calculate the homeostatic model of insulin resistance (HOMA2-IR) [glycemia (mmol/l) × insulinemia (µU/ml)/22,5] and using glucose and insulin values during OGTT to calculate the Matsuda index of insulin sensitivity (ISI Matsuda) (10,000/glucose (mg/dl) × insulin (µU/ml) × glucose mean × insulin mean)^15,16^. A composite measure of β-cell function relative to insulin sensitivity, assessed by Oral Disposition Index (DIo), was calculated as (ΔInsulin_0–30_/ΔGlucose_0–_30) × (1/fasting insulin). The trapezoidal method was used for calculation of the areas under the curves for insulin (AUC_2hInsulin_) and glucose (AUC_2hglucose_)^17^.

The Visceral Adiposity Index (VAI) was calculated according to gender, where TG is triglycerides expressed in mmol/l and HDL is HDL-cholesterol levels expressed in mmol/l^18^:

$$ {\text{Males}}\;{\text{VAI}} = \left[ {{\text{WC}}/{39}.{68} + \left( {{1}.{88} \times {\text{BMI}}} \right)} \right] \times \left( {{\text{TG}}/{1}.0{3}} \right) \times \left( {{1}.{31}/{\text{HDL}}} \right); $$

$$ {\text{Females}},\;{\text{VAI}} = \left[ {{\text{WC}}/{36}.{58} + \left( {{1}.{89} \times {\text{BMI}}} \right)} \right] \times \left( {{\text{TG}}/0.{81}} \right) \times \left( {{1}.{52}/{\text{HDL}}} \right). $$

### Hormone and biochemical assays

Serum insulin, glycaemia and lipid levels were measured by ELISA (DRG instruments GmbH, Germany) as previously reported^7^. The normal insulin range was 5–19 UI/ml. LDL cholesterol was measured using the Friedewald formula [total cholesterol − (HDL + (TG/5)]. HbA1c was determined by High Pressure Liquid Chromatography (HPLC) with ion-exchange resin (HA8121, Hi-AutoA1c, Menarini Italy). The conversion factors for the International System (SI) were the following: glucose mg/dl versus mmol/l: 0.0555, insulin mUI/ml versus pmol/l: 6.945, total and HDL cholesterol mg/dl versus mmol/l: 0.0259, triglycerides mg/dl versus mmol/l: 0.0113.

### Statistical analysis

SPSS version 17 was used for data analysis as previously reported^7^. Baseline characteristics were presented as mean ± SD for continuous variables; rates and proportions were calculated for categorical data. Normality of distribution for quantitative data was assessed by the Shapiro–Wilk test. The differences between patients with NGT and prediabetes/DM were detected by Student’s t test for continuous variables and by the chi-square test for categorical variables. Crude odds ratios (OR) and their 95% confidence interval (CI) for the association of diabetes with potential risk factors in SAI were calculated by univariate analysis. The correction for multiple comparisons was performed by Bonferroni post-hoc test. Adjusted ORs were calculated by stepwise logistic regression analysis to identify factors independently associated with development of diabetes. Only factors significantly associated with diabetes mellitus by univariate analysis were included in the logistic regression analysis. A receiver operating characteristic (ROC) analysis was performed to investigate the diagnostic ability of significantly associated risk factors to predict diabetes, except for parameters which were already standardized such as WC. The ROC curve is plotted as sensitivity versus 1‐specificity. The area under the ROC curve (AUC) was estimated to measure the overall performance of the predictive factors for diabetes mellitus. A *p* value of < 0.05 was considered statistically significant.

## Results

The characteristics of the patients with SAI and the other endocrine deficiency combinations are shown in Table 1. Of 102 patients with SAI, 42 were classified as having a defect in glucose metabolism, such as prediabetes or diabetes mellitus. Higher frequency of family history of diabetes (*p* = 0.002) was found in patients with prediabetes/DM than NGT (Table 1). Patients with prediabetes/DM had higher BMI (*p* < 0.001), WC (*p* < 0.001), total cholesterol (*p* = 0.012), LDL-cholesterol (*p* = 0.004), TG (*p* = 0.031), fasting glucose (*p* = 0.002), fasting insulin (*p* = 0.035), glutamate-pyruvate transaminase (GPT) (*p* = 0.018), HOMA-IR (*p* = 0.039), AUC_2h glucose_ (*p* = 0.001), AUC_2 h insulin_ (*p* = 0.002), HbA1c (*p* < 0.001), VAI (*p* = 0.038) and lower ISI-Matsuda (*p* = 0.008) and DIo (*p* < 0.001) than patients with NGT (Table 1).

**Table 1:** Clinical and biochemical features of all patients with secondary adrenal insufficiency divided into normal glucose tolerance (NGT) and glucose metabolism disorders (diabetes mellitus, DM, and prediabetes, preDM).

	NGT No 60	Pre DM/DM No 42	*p*
	Subjects (%)	Subjects (%)	
Male	23 (38.3%)	12 (28.5%)	0.243
Female	37 (61.6%)	30 (71.4%)	
Family history of diabetes	11 (18.3%)	17 (40.4%)	0.002
Hypertriglyceridemia	18 (29%)	10 (45.5%)	0.128
Hypercholesterolemia	5 (8.3%)	9 (21.4%)	0.059
Visceral obesity	50 (83.3%)	32 (76.1%)	0.370
Arterial hypertension	7 (11.6%)	9 (21.4%)	0.195
Hypothyroidism	44 (73.3%)	35 (83.3%)	0.236
Hypogonadism	39 (65%)	25 (59.5%)	0.573
GH deficiency	25 (41.6%)	18 (42.8%)	0.904
Hydrocortisone replacement therapy	32 (53.3%)	18 (42.8%)	0.298
Cortisone acetate replacement therapy	28 (46.6%)	24 (57.1%)	0.347
High steroid replacement therapy	25 (41.6%)	24 (57.1%)	0.156

	NGT No 60	Pre DM/DM No 42	*p*
	Mean ± SD	Mean ± SD	
Age (years)	48.7 ± 12.3	49.8 ± 12.7	0.666
Duration of replacement therapy (years)	13.3 ± 9.88	18.1 ± 13.4	0.043
BMI (Kg/m^2^)	24.9 ± 4.06	29.1 ± 5.48	< 0.001
WC (cm)	92.5 ± 12.2	101.5 ± 14.5	< 0.001
Dose of cortisone acetate	30.9 ± 9.82	37.6 ± 13.1	0.063
Dose of hydrocortisone	17.3 ± 4.91	19.1 ± 6.24	0.248
Total cholesterol (mmol/l)	4.93 ± 0.85	5.66 ± 1.15	0.012
HDL cholesterol (mmol/l)	1.53 ± 0.49	1.49 ± 0.53	0.740
LDL cholesterol mmol/l)	2.65 ± 0.88	3.24 ± 0.87	0.004
Triglycerides (mmol/l)	1.37 ± 0.72	1.71 ± 0.64	0.031
Fasting glucose (mmol/l)	4.46 ± 0.51	7.13 ± 4.65	0.002
Fasting insulin (UI/ml)	7.54 ± 4.41	12.7 ± 9.23	0.035
GOT (U/L)	19.9 ± 9.07	24.2 ± 12.2	0.178
GPT (U/L)	17.5 ± 8.95	27.5 ± 19.2	0.018
Homa 2-IR	1.46 ± 0.92	2.47 ± 1.76	0.039
AUC_2h glucose_	12,804.1 ± 2717.5	17,758.2 ± 4666.9	0.001
AUC_2h insulin_	6828.5 ± 5382.1	14,294.3 ± 7824.4	0.002
ISI Matsuda	10.8 ± 9.55	2.57 ± 1.25	0.008
Oral Disposition Index (DIo)*	6.39 ± 2.47	1.95 ± 1.47	< 0.001
HbA1c (%)	5.33 ± 0.43	6.99 ± 1.96	< 0.001
VAI	1.76 ± 1.36	2.54 ± 1.28	0.038

After, we compared patients with DM on HC and cortisone acetate therapy and observed that patients treated with HC had lower TG (*p* = 0.007) and VAI (*p* = 0.010) and higher HDL-cholesterol (*p* = 0.005) than those treated with cortisone acetate (Table 2).

**Table 2:** Hydrocortisone versus cortisone acetate replacement therapy in patients with diabetes mellitus (DM)/prediabetes.

DM/prediabetes No 42			
	Hydrocortisone No 18	Cortisone acetate No 24	*p*
	Mean ± SD	Mean ± SD	
Age (years)	46.5 ± 13.5	53.5 ± 10.3	0.087
Duration of disease (years)	17.4 ± 14.9	18.8 ± 12.2	0.757
BMI (Kg/m^2^)	27.4 ± 5.11	30.1 ± 5.54	0.141
WC (cm)	97.4 ± 11.6	103.5 ± 15.8	0.203

DM/prediabetes No 42			
	Hydrocortisone No 18	Cortisone acetate No 24	
	Subjects (%)	Subjects (%)	
Male	7 (38.8%)	6 (25%)	0.344
Female	11 (61.1%)	18 (75%)	
Family history of Diabetes	5 (27.7%)	12 (50%)	0.149
Arterial hypertension	5 (27.7%)	8 (33.3%)	0.701
Hypertriglyceridemia	10 (55.5%)	18 (75%)	0.190
Hypercholesterolemia	3 (16.6%)	6 (25%)	0.516
Visceral obesity	16 (88.8%)	16 (66.6%)	0.099

DM/prediabetes No 42			
	Hydrocortisone No 18	Cortisone acetate No 24	*p*
	Mean ± SD	Mean ± SD	
**Metabolic parameters**			
Total Cholesterol (mmol/l)	5.65 ± 1.41	5.64 ± 0.96	0.979
HDL Cholesterol (mmol/l)	1.78 ± 0.57	1.26 ± 0.31	0.005
LDL cholesterol mmol/l)	3.06 ± 0.97	3.39 ± 0.87	0.338
Triglycerides (mmol/l)	1.43 ± 0.53	1.99 ± 0.51	0.007
Fasting glucose (mmol/l)	5.61 ± 2.51	8.41 ± 5.63	0.085
Fasting insulin (UI/ml)	10.7 ± 9.39	14.2 ± 9.05	0.514
GOT (U/L)	21.3 ± 16.9	25.9 ± 6.57	0.398
GPT (U/L)	25.6 ± 17.05	28.4 ± 9.99	0.687
Homa 2-IR	2.19 ± 1.56	2.99 ± 2.15	0.384
HbA1c (%)	6.87 ± 1.10	7.78 ± 1.76	0.070
VAI	1.85 ± 0.94	3.18 ± 1.25	0.010

The comparison between patients on high and low doses of steroids did not show any significant differences (Table 3).

**Table 3:** High versus low doses of steroid replacement therapy in patients with diabetes mellitus (DM)/prediabetes.

DM/prediabetes No 42			
	Low doses No 18	High doses No 24	*p*
	Mean ± SD	Mean ± SD	
Age (years)	48.1 ± 14.1	53.5 ± 11.1	0.167
Duration of disease (years)	16.2 ± 17.9	19.3 ± 9.31	0.472
BMI (Kg/m^2^)	27.6 ± 6.65	29.5 ± 4.55	0.273
WC (cm)	97.9 ± 14.7	101 ± 14.2	0.505

DM/prediabetes No 42			
	Low doses No 18	High doses No 24	
	Subjects (%)	Subjects (%)	
Male	8 (44.4%)	5 (20.8%)	0.174
Female	10 (55.6%)	19 (79.2%)	
Family history of Diabetes	7 (38.8%)	10 (41.6%)	0.545
Arterial hypertension	3 (16.7%)	10 (41.6%)	0.184
Hypertriglyceridemia	5 (27.8%)	13 (54.1%)	0.237
Hypercholesterolemia	3 (16.7%)	6 (25%)	0.275
Visceral obesity	10 (55.6%)	22 (91.7%)	0.158
Hydrocortisone replacement therapy	10 (55.6%)	8 (33.3%)	0.470
Cortisone acetate replacement therapy	8 (44.4%)	16 (66.7%)	0.174

DM/prediabetes No 42			
	Low doses No 18	High doses No 24	*p*
	Mean ± SD	Mean ± SD	
**Metabolic parameters**			
Total cholesterol (mmol/l)	5.47 ± 0.88	5.81 ± 1.37	0.419
HDL cholesterol (mmol/l)	1.44 ± 0.52	1.46 ± 0.56	0.940
LDL cholesterol mmol/l)	3.18 ± 0.73	3.28 ± 1.06	0.311
Triglycerides (mmol/l)	1.61 ± 0.47	1.73 ± 0.65	0.744
Fasting glucose (mmol/l)	5.58 ± 4.16	7.57 ± 4.51	0.174
Fasting insulin (UI/ml)	9.23 ± 6.09	14.1 ± 9.8	0.294
GOT (U/L)	25.6 ± 19.1	21.8 ± 7.71	0.601
GPT (U/L)	30.3 ± 15.6	23.2 ± 10.5	0.542
Homa 2-IR	2.94 ± 2.31	1.95 ± 1.21	0.216
HbA1c (%)	6.71 ± 1.47	7.33 ± 1.66	0.165
VAI	2.41 ± 0.82	3.06 ± 1.71	0.199

**Figure 1:**
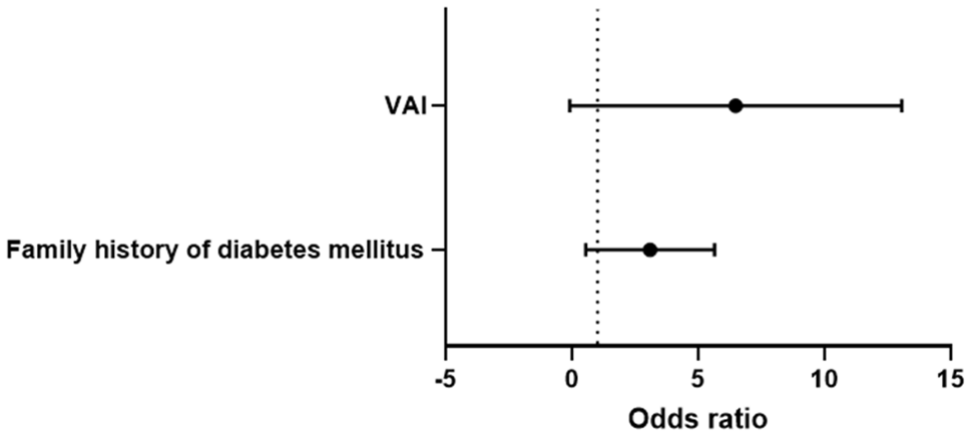
A Forest Plot showing odds ratio values and 95% confidence intervals for: DM and SAI, adjusted by BMI and dose of cortisone acetate. The x-axis represents the odds ratio (circles) and 95% confidence intervals (whiskers). The dashed vertical line indicates an OR value of 1.

Univariate analysis was performed by using the abovementioned risk factors as potential predictors for diabetes mellitus (crude odd ratios) (Table 4). A ROC curve was constructed, and a prediction model was established with a moderately robust power (AUC = 0.73) to predict diabetes mellitus in patients with SAI (except for WC). Our model after Bonferroni correction post-hoc test demonstrates that BMI (*p* = 0.046), dose of cortisone acetate (*p* = 0.034), VAI (*p* = 0.001) and family history of diabetes (*p* = 0.004) were statistically significant factors for predicting the development of diabetes mellitus in SAI. At multivariate analysis, family history of diabetes mellitus (*p* = 0.005) and VAI (*p* = 0.001) were found to be predictors of diabetes mellitus (adjusted odd ratios) (Fig. 1). A *p* value < 0.005 was statistically significant.

**Table 4:** Risk factors associated with glucose metabolism disorders (diabetes mellitus, DM, and prediabetes, preDM) versus normal glucose tolerance (NGT).

Variable	DM/preDM (No = 42)	NGT (No = 60)	Crude OR (95% CI)
**BMI**			
≤ 22.8 kg/m^2^	3 (7.7%)	22 (36.6%)	1
> 22.8 kg/m^2^	39 (92.3%)	38 (63.4%)	6.93 (1.51–31.7)
**Duration of disease**			
< 16 years	16 (38.5%)	42 (69.3%)	1
≥ 16 years	26 (61.5%)	18 (30.7%)	3.61 (1.42–9.16)
**VAI**			
≤ 1.74	11 (4%)	38 (63.9%)	1
> 1.74	31 (75%)	22 (36.1%)	5.31 (1.41–19.8)
**Total cholesterol**			
≤ 6.01 mmol/L	28 (66.7%)	52(87.3%)	1
> 6.01 mmol/L	14 (33.3%)	8 (12.7%)	3.43 (1.06–11.1)
**LDL-cholesterol**			
≤ 2.36 mmol/L	6 (14.3%)	17 (28.8%)	1
> 2.36 mmol/L	36 (85.7%)	43 (71.2%)	2.42 (0.63–9.32)
**Triglycerides**			
≤ 1.62 mmol/L	18 (42.9%)	42 (69.8%)	1
> 1.62 mmol/L	24 (57.1%)	18 (30.2%)	3.08 (1.11–8.64)
**GPT**			
≤ 28 U/L	26 (62.5%)	53 (88.6%)	1
> 28 U/L	16 (37.5%)	7 (11.4%)	4.65 (1.08–19.8)
**Familial history of diabetes**			
No	19 (46.6%)	49 (81.3%)	1
Yes	23 (53.8%)	11 (18.7%)	2.5 (1.13–6.94)
Waist circumference females*			
< 88 cm	9 (30%)	30 (81%)	1
≥ 88 cm	21 (70%)	7 (19%)	4.75 (1.51–14.9)
Waist circumference males**			
< 102 cm	3 (25%)	19 (82.6%)	1
≥ 102 cm	9 (75%)	4 (17.4%)	11.8 (1.19–98.4)
Dose of cortisone acetate***			
≤ 25 mg/day	8 (33.3%)	23 (58.9%)	1
> 25 mg/day	16 (66.7%)	16 (41.1%)	10.5 (2.68–41.1)

*Calculated in a sample of 30 females with DM/preDM and 37 with NGT.

**Calculated in a sample of 12 males with DM/preDM and 23 with NGT.

***Calculated in a sample of 24 with DM/preDM and 39 with NGT.

## Discussion

In this study we aim to evaluate the prevalence of DM and to identify which factors can predict development of DM in patients with SAI. Our study confirms the high frequency of DM in patients with SAI on conventional steroid replacement therapy and a significant association with VAI and family history of diabetes.

Glucocorticoids (GCs) have a strong impact on glucose homeostasis by two mechanisms: favouring peripheral insulin resistance in liver, skeletal muscle and adipose tissue and reducing insulin secretion^19,20^. Chronic excessive treatment with GCs exposes subjects to the increase in body fat mass with higher visceral abdominal to peripheral subcutaneous fat distribution^21,22^.

Cortisone acetate and HC have different pharmacokinetics. Indeed, HC is a biologically active GC, while cortisone acetate requires conversion to cortisol by the enzyme 11-β-hydroxysteroid dehydrogenase type 1 to become active, resulting in a delayed peak of serum concentration compared to HC.

Several studies reported the metabolic effects of conventional steroids in AI. In a study conducted on 2424 patients with SAI, doses of prednisolone or HC of more than 20 mg/day were associated with higher lipids values than cortisone acetate, without differences in the comparison of HC with prednisolone^8^. In another study, treatment with prednisolone was associated with higher total and LDL-cholesterol than HC^23^. By contrast, Smith et al. compared patients treated with HC thrice daily with patients on prednisolone once daily without significant differences in hematological parameters in the two groups^24^.

Interestingly, the switch from cortisone acetate to HC at the conversion dose of 4:5 was recently reported to be associated with increase in body weight, total fat mass, WC and HbA1c, likely due to higher potency of HC than cortisone acetate^25^. We recently showed that long-term treatment with conventional steroids, HC and cortisone acetate is associated with an increase in BMI, WC, systolic blood pressure, LDL-cholesterol, HbA1c and Framingham risk score^26^.

High doses of cortisone acetate over 30 mg/day and HC over 20–25 mg/day were found to be associated with unfavourable metabolic phenotype and increased cardiovascular risk factors than lower doses^3,8,27^. However, conflicting data are reported on the use of lower rather than higher doses of HC. The decrease of HC dose from 30 to 15 mg/day has been shown to be neutral in body weight and cardiovascular function in a small cohort of patients with SAI^10^. By contrast, another study showed a significant decrease in body weight and lipid profile without any effects on insulin and fasting glucose levels after reducing HC doses^11^. Higher conventional GC doses did not impact on insulin sensitivity and secretion, while were associated with lower fasting augmentation index and reactive hyperaemia index in patients with SAI^12^. A large French study reported high lipids and blood pressure levels in patients with PAI and SAI treated with high doses of HC^28^. Similarly, another study reported lower arterial stiffness and a more physiological nocturnal blood pressure dip in patients treated with low doses of HC without differences in insulin sensitivity^29^.

In the current study we did not find any significant association between diabetes mellitus and steroid replacement therapy, suggesting that other factors more than the dose or the type of steroids can impact on diabetes mellitus. Indeed, we found that family history of DM predisposes to metabolic derangement. It is noteworthy that DM is a polygenic disease and among all the genetic factors described, the presence of relatives affected by the disease remains one of the most important determinants in predicting the onset of disease^30^.

Lastly, we also found that high VAI was associated with DM. VAI is a gender-specific mathematical formula based on BMI, WC, HDL-cholesterol and triglycerides, highly correlated with visceral adiposity. It is associated with cardiometabolic risk in the adult population more markedly than BMI and WC. In addition, recently many studies have reported that VAI could be used as method to predict DM even though no specific cut-off points have been identified^31^.

The study has some limitations. First, the sample evaluated is quite heterogeneous and the doses of HC were significantly lower than those used in other studies. Second, few patients (13 out of 42) were treated with anti-hypertensive agents, calcium channel blockers and ARBs which could partly affect glucose metabolism^32^. Lastly, we did not assay circulating catecholamine levels which play a significant role in blood glucose regulation. Indeed, impaired epinephrine secretion in response to insulin-induced hypoglycaemia has reported in patients with SAI^33,34^. The strength of the study is that, to our knowledge, no previous reports have suggested the predictive factors for diabetes development in patients with SAI.

In conclusion, the results of our study suggest that DM in patients with SAI is quite frequent. Factors such as family history of DM and VAI could be predictors of development of DM in patients with SAI, while other factors such as the dose and the type of replacement steroid treatment do not impact on DM.

However, further controlled larger studies are required to confirm out preliminary results.
